# Measuring Community and Home Participation and Environmental Factors in Children with Cerebral Palsy

**DOI:** 10.3390/pediatric17010017

**Published:** 2025-02-07

**Authors:** Turki Aljuhani, Shaden A. Alzahrani, Abeer M. Aldosary, Lana A. Alzamil, Rakan K. Alshehri, Afnan S. Gmmash, Reem A. Albesher

**Affiliations:** 1 Department of Occupational Therapy, College of Applied Medical Sciences, King Saud Bin Abdulaziz University for Health Sciences, Riyadh 114881, Saudi Arabia; alzhrani300@ksau-hs.edu.sa (S.A.A.); aldosary306@ksau-hs.edu.sa (A.M.A.); 2 King Abdullah International Medical Research Center, Riyadh 11481, Saudi Arabia; zamill@ksau-hs.edu.sa (L.A.A.); alsuqalira@ngha.med.sa (R.K.A.); 3 Research and Innovation Unit, College of Applied Medical Sciences, King Saud Bin Abdulaziz University for Health Sciences, Riyadh 11481, Saudi Arabia; 4 Department of Rehabilitation, King Abdullah Specialized Children’s Hospital, Ministry of National Guard Health Affairs, Riyadh 11426, Saudi Arabia; 5 Department of Physical Therapy, Faculty of Medical Rehabilitation Sciences, King Abdulaziz University, Jeddah 21589, Saudi Arabia; asgmmsh@kau.edu.sa; 6 Department of Rehabilitation Sciences, College of Health and Rehabilitation Sciences, Princess Nourah Bint Abdulrahman University, Riyadh 11671, Saudi Arabia; raalbesher@pnu.edu.sa

**Keywords:** participation, cerebral palsy, community, children, environmental factors

## Abstract

**Background/Objectives**: Children with cerebral palsy (CP) are reported to have lower rates of participation in community and home tasks than typically developing children. Little is known about the participation levels of children with CP and the environmental factors influencing their participation in the community within the Saudi context. This study aimed to determine the degree of participation in community and home tasks in children with CP in Saudi Arabia, as well as the level of support received, and the obstacles faced by children in this context. **Methods**: Parents of 5–16-year-old children with CP (*n* = 50) completed the Participation and Environment Measure for Children and Youth (PEM-CY), and their scores were compared with those of typically developing children of the same ages (*n* = 50). This study was conducted in a hospital setting. Analyses were performed using multivariate logistic regression analyses, controlling for potential confounders. **Results**: Participation in community activities as well as home activities was reported to be significantly lower in children with CP compared to their typically developing peers in multiple domains (*p* < 0.05). Additionally, physical, cognitive, and social demands were identified as factors that made community participation more difficult for children with CP (*p* = 0.002, *p* = 0.017, and *p* = 0.029 for physical, cognitive, and social demands, respectively). The availability of personal transportation, programs, services, and financial support were identified by parents as the factors that lowered community participation levels in children with CP (*p* > 0.005). **Conclusions**: Physical, cognitive, and social demands are the main obstacles to participation for children with CP. A lack of home supplies and financial support lowered the participation of children with CP. Recognizing environmental barriers as well as providing individualized practical solutions in collaboration with the families of children with CP can assist in developing meaningful participation in community and home activities for children with CP.

## 1. Introduction

Children of all ages require active participation in home and community-based activities to promote their health and well-being. Active participation involves children’s engagement in various recreational, leisure, and academic activities that promote their development. Meaningful community participation and purposeful engagement with peers provide children with the necessary prerequisite skills for psychosocial and physical growth [1]. The International Classification of Functioning, Disability and Health framework (ICF) illustrates that personal and environmental factors influence children’s rates of participation. Personal and environmental factors can be either facilitators or barriers for individuals with disabilities. Facilitators are any personal or environmental elements that can help an individual participate in their community, while barriers are those which can hinder the individual’s ability to participate in the community, such as poverty vs. wealth and urban vs. rural settings [2].

Certain health conditions restrict children’s physical abilities, limiting their community participation [2]. Over the past two decades, the participation of children with disabilities in the community has received increasing attention, with more studies aimed at measuring their participation in the community and identifying the need for change. This is partly because participation is a key component of the ICF framework, in which participation is defined as “involvement in a life situation” [2]. Lack of participation can therefore lead to children with disabilities encountering difficulties which restrict their learning and reduce their participation compared to that of typically developing children [3,4]. Children with cerebral palsy (CP) commonly have multiple impairments that limit their speech and intellectual capacities, in addition to their physical disabilities, which further restrict their participation at home and in the community [5]. Studies have reported that the participation of children with CP in home and community settings is lower than that of their typically developing peers [6]. Children with CP do not often participate in recreational activities, and their personal interests and hobbies must be considered in intervention plans, as they may influence their participation [7,8].

The participation of children with CP in community-based activities is affected by the severity of their impairments and their family factors [9]. Gross motor function measured using the Gross Motor Function Measure Classification Scale (GMFCS) can significantly impact community accessibility and influence children’s participation [10,11]. In addition, age was found to be a factor affecting children’s participation, as older children have higher functional abilities than younger children [11].

In addition, typically developing children develop more friendships than children with disabilities [12]. Developing meaningful and healthy friendships can curtail the effects of bullying, adult depression, and emotional abuse [13]. Certain family characteristics and preferences for leisure activities can alter children’s decisions regarding the type of activities in which they participate [14]. Living in a rural or urban area [15] and provisions of emotional, financial, and social support from families and communities can facilitate or hinder children’s community participation [14,16].

In the Middle East, children with disabilities might experience additional barriers affecting their participation and inclusion in age-appropriate activities such as attending school, competing in sports, and playing on playgrounds [17]. Some of these barriers include a lack of safety measures, ramps, qualified educators, and special equipment [17]. Although noticeable advancements have been made in Saudi Arabia over the past few years concerning the inclusion of children with disabilities, challenges remain [18]. More efforts are needed to support educators in providing optimal services to reinforce the inclusion of children with disabilities in mainstream activities [18,19]. In a relatively new systematic review, which identifies Arabic patient-reported measures of activity and participation, the evidence suggests a need for more culturally adapted tools that assess the participation of children in the Arabic speaking population [20]. Assessing the participation of children with disabilities is vital and must be emphasized to identify factors that hinder children’s holistic development, and their progress should be monitored in therapy. This in turn should facilitate the inclusion of children with disabilities in the community. In the Middle East, a clear call for the social inclusion of children with developmental disability has been voiced recently [21].

Due to the limited number of studies that investigate children’s participation in the Middle East and Saudi Arabia in particular, our study intended to improve our understanding in comparison with current reported levels of participation in other parts of the word. This understanding will add in inform healthcare planning, enhance children health, identify barriers and facilitators in the community and monitor policy and advocacy efforts. Thus, our study aimed to (i) examine the degree to which children with CP participation in their home and community-based settings and (ii) investigate the factors associated with their participation by using the Participation and Environment Measure for Children and Youth (PEM-CY). The results of this study add to the evidence on the participation of children with disabilities in activities at different recreational, leisure, and academic settings.

## 2. Materials and Methods

### 2.1. Design

This prospective study was conducted at King Abdullah Specialized Children’s Hospital (KASCH) in Riyadh, Saudi Arabia from September 2022 to April 2023. A convenience sampling was used to recruit participants via chart review from KASCH outpatient clinics, which included parents of 50 children aged 5 to 16 years with CP diagnoses and involvement in the community. Fifty typically developing children’s parents in the same age group were contacted and agreed to participate in the study. Parents were initially contacted via phone, and an email was sent with a link to online questionnaires that included the Arabic version of the PEM-CY questionnaire. Parents had the option to complete the survey on their own time, thus reducing the potential respondent burden. Parents completed all PEM-CY questionnaires (home and community participation). Ethical approval was obtained from King Abdullah International Medical Research Center (KAIMRC) (Reference No. 071/05). Parents consented to the study prior to enrollement. A post hoc power analysis was conducted to evaluate whether the sample size (*n* = 100, 50 CP children and 50 normal children) was adequate to detect significant differences between the two groups. Assuming a 30% difference in proportions and an alpha level of 0.05, the statistical power was calculated to be 81%, confirming the sample size was sufficient to detect meaningful differences.

### 2.2. Outcome Measures

The PEM-CY is a questionnaire that asks parents about their children’s frequency and involvement in activities in three domains: home, school, and community [22]. The PEM-CY has moderate to good internal consistency and reliability and showed significant differences between groups with and without disability in all domains [22]. The PEM-CY home questionnaire contained a total of 22 questions, while the environment section has 26 questions. This study focused on the home and community domains, which inquire about the level of participation, involvement, and desire to change the community and home activities. In addition, the questionnaire assessed support, barriers, and resources for parents in the home and community environments. The parents were asked whether the child participates in certain home and community activities, the child’s level of involvement, and the parent’s desire to change their child’s participation levels [7]. Parents were also asked about certain environmental factors that might help or make it more difficult for their children to participate in home and community activities, such as physical, sensory, cognitive, and social demands, as well as money, supply, and information.

In this study, parents rated their children’s frequency of participation as participating or never participating. For each task that children participated in, parents rated the participation frequency as: daily, weekly, or monthly. Parents rated their children as having either a high or low level of involvement in the task. In addition, they reported a desire to change the child’s level of participation with “yes” or “no”. Furthermore, parents reported support or barriers in the home and community as: usually helpful, usually making it hard, or not an issue). Finally, the parents rated accessibility and availability as needed or not with a “yes” or “no”.

### 2.3. Statistical Analysis

For descriptive analyses, responses for the demographic characteristics, level of participation, support, barriers, and availability of resources at home and community variables are presented as frequency and percentages. Differences in demographic characteristics between two groups were tested using chi-square test for categorical variables and *t*-test for continuous variables. Multivariate logistic regression analyses were used to compare children with CP with their typically developing peers, rating their level of participation, involvement, desire to change, home and community environmental factors (barriers or support), and availability of resources. Adjust for potential confounders, including age, gender, GMFCS level, mother’s education and father’s education, and calculate adjusted odds ratios (AORs) with a 95% confidence interval (CI) for all variables. Multicollinearity among the potential confounders was assessed using Spearman correlation coefficients and Chi-Square tests. All correlations were below 0.1 with no statistically significant relationships (*p* > 0.05), confirming the independence of the potential confounders and ensuring the robustness of the logistic regression models. The Akaike Information Criterion (AIC) and Bayesian Information Criterion (BIC) were used to evaluate model fit, with AIC values ranging from 71.270 to 138.070 and BIC values ranging from 94.308 to 168.842, indicating adequate fit for the analyzed data. Receiver Operating Characteristic (ROC) curves were generated to evaluate the logistic regression models. The Area Under the Curve (AUC) values ranged from 0.787 to 0.815, indicating good to very good model performance. Given the consistency in analysis methodology and variable structure, similar performance is expected across all models. All analyses were conducted using Stata version 15, (Statacorp, College Station, TX, USA).

## 3. Results

### 3.1. Participant Characteristics

This study included parents of 100 children, 50 with a confirmed diagnosis of CP and 50 typically developing children. Parents of children with CP were recruited from the outpatient clinic of King Abdullah Specialized Children’s Hospital. The clinical and demographic characteristics of the children are presented in Table 1. The mean age of the children was 10.56 (±2.314) years; 56% of the children were male. The number of children diagnosed with spastic diplegia was the highest (36%), followed by those diagnosed with spastic quadriplegia 30%. As for the GMFCS level, level II had the highest share of children with CP (38%), meaning they can walk indoors and outdoors and climb stairs using a railing, but are unable to run or jump, while 36% were in level III, where they require an assistive mobility device indoors and outdoors. Unfortunately, 21 of the 50 children with CP did not enroll in integrated schools, according to their parents. This led our research team to exclude the school domain of the PEM-CY from our results.

Furthermore, the majority of children’s mothers had high levels of education (21%), while the majority of fathers had high school education (50%). Children with CP were similar to typically developing children. However, there was a significant difference between the two groups in the mother’s education level, and type of school (*p* = 0.002, *p* ≤ 0.001). The difference in the type of schools was mainly noted with children with CP enrolling in an inclusion school (12%) or not enrolled in any school (42%).

### 3.2. Community Participation Frequency, Involvement, and Desire to Change the Current Level of Participation

**Figure 1:**
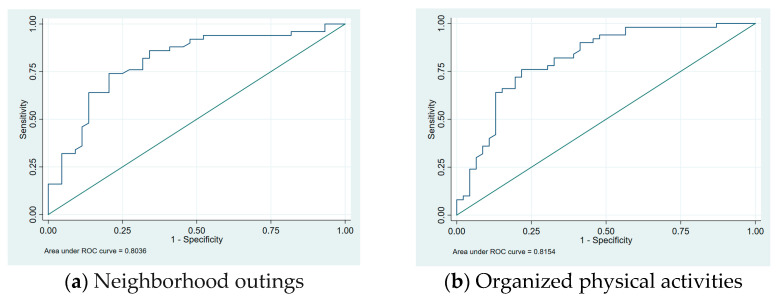
Community activities data visualization ROC curve.

A comparison between PEM-CY community participation frequency ratings of parents of children with CP and those of typically developing children is shown in Table 2. Parents of children with CP reported less to no participation in activities that required physical demands. There were significant differences in the following five community items between the two groups: neighborhood outings (AOR = 0.105, CI: 0.020–0.539, *p* = 0.007 weekly; AOR = 0.167, CI: 0.035–0.799, *p* = 0.0025 monthly), organized physical activities (AOR = 0.024, CI: 0.005–0.671, *p* = 0.002 daily; AOR = 0.184, CI: 0.054–0.628, *p* = 0.007 weekly; AOR = 0.059, CI: 0.002–0.256, *p* = 0.022 monthly), unsupported classes (AOR = 0.132, CI: 0.039–0.480, *p* = 0.002 weekly; AOR = 0.180, CI: 0.032–0.994, *p* = 0.049 monthly), religious activities (AOR = 0.149, CI: 0.048–0.463, *p* = 0.001 daily), and getting together with friends in the community (AOR = 0.047, CI: 0.004–0.619, *p* = 0.020 daily; AOR = 0.071, CI: 0.007–0.688, *p* = 0.022 weekly; AOR = 0.061, CI: 0.005–0.699, *p* = 0.025 monthly). Figure 1 showed the data visualization for two community activities that were significantly different between the two groups (neighborhood outings and organized physical activities).

Additionally, the involvement of children whose parents reported participating in the community was compared (Supplementary Table S1). Parents of children with CP reported significantly lower involvement of their children in organized physical activities (AOR = 0.145, CI: 0.049–0.431, *p* = 0.001), unsupported class activities (AOR = 0.238, CI: 0.088–0.649, *p* = 0.005) and religion or spiritual gathering activities (AOR = 0.171, CI: 0.061–0.481, *p* = 0.001) than that of typically developing children. Lastly, parents of children with CP reported a significant desire to change two of the mentioned community activities: religion or spiritual gathering activities (AOR = 3.227, CI: 1.218–8.550, *p* = 0.018) and getting together with friends in the community (AOR = 3.063, CI: 1.159–8.100, *p* = 0.024). (Supplementary Table S2).

### 3.3. Home Participation Frequency, Involvement, and Desire to Change the Current Level of Participation

**Figure 2:**
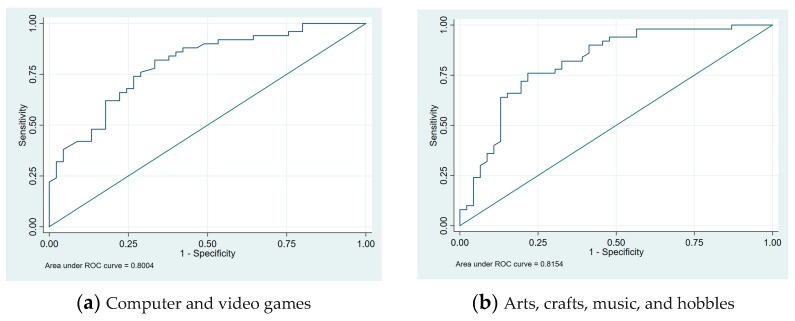
Home activities data visualization ROC curve.

A comparison between the reports of parents of children with CP and typically developing children on PEM-CY home participation frequency is shown in Table 3. Parents of children with CP reported less or no participation in activities that required physical demands. There were significant differences in the three home activities in the two groups: hobbies related to computers and video games (AOR = 14.371, CI: 1.468–140.730, *p* = 0.022 monthly), hobbies related to art and music (AOR = 16.420, CI: 3.156–85.443, *p* = 0.001 monthly), and getting together with family members (AOR = 4.598, CI: 1.138–18.575, *p* = 0.032 monthly). Figure 2 shows the data visualization for two home activities that were significantly different between the two groups (computer and video games and art, crafts, music, and hobbies).

Additionally, the involvement of children whose parents reported participating at home was compared (Supplementary Table S3). Parents of children with CP reported significantly lower involvement of their children in the same three home activities mentioned above plus the completing household chores. Parents of children with CP also reported a significant desire to change participation levels in two home activities: hobbies related to art and music (AOR = 3.951, CI: 1.538–10.146, *p* = 0.004) and personal care management (AOR = 4.544, CI: 1.646–12.539, *p* = 0.003) (Supplementary Table S4).

### 3.4. Home Environment Perceived Availability and Adequacy of Resources

**Figure 3:**
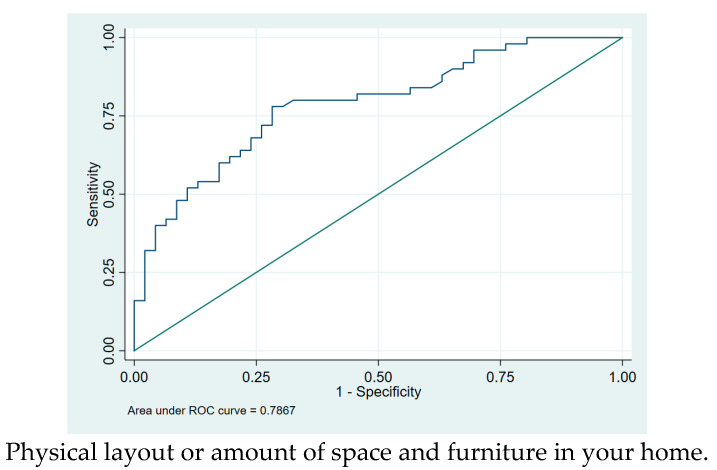
Supportiveness of the home environment data visualization ROC curve.

Parents’ ratings of home factors in the home environment are reported in Table 4. Parents of children with CP reported significantly more difficulties in the following home environment compared with the parents of typically developing children: physically demanding activities (usually helpful: AOR = 0.166, CI: 0.052–0.527, *p* = 0.002), cognitively demanding activities (usually helpful: AOR = 0.116, CI: 0.020–0.685, *p* = 0.017), and social demands (usually helpful: AOR = 0.075, CI: 0.007–0.771, *p* = 0.029). Figure 3 shows the data visualization for the physical layout in the home which was significant between the two groups.

Many parents of children with CP reported that services and supplies were usually unavailable or inadequate to support their child’s participation at home (Supplementary Table S5). Over 50% of the parents of children with CP reported that supplies at home were not sufficient to engage their children in home participation tasks. Only 30% of the parents of children with CP identified money as a resource that was usually unavailable or adequate to support home participation. Analysis showed significant differences between children with CP and typically developing children’s cohort availability and adequacy of supplies (AOR = 0.018, CI: 0.002–0.170, *p* ≤ 0.001) and money (AOR = 0.067, CI: 0.019–0.230, *p* ≤ 0.001) to support children with CP’s participation at home.

### 3.5. Community Environment Perceived Availability and Adequacy of Resources

Parent ratings of the environmental factors in the community that influenced their children’s participation are reported in Table 5. Parents of children with CP reported significantly more difficulties in the following community environment in only one domain compared with that of typically developing children: safety in society (usually made harder: AOR = 0.261, CI: 0.077–0.893, *p* = 0.032). Most parents of children with CP reported that services and supplies were usually not available or were inadequate to support their children’s participation in the community (Supplementary Table S6). More than 50% of the parents of children with CP reported that personal transportation was either unavailable or was difficult to obtain in the community (AOR = 0.220, CI: 0.796–0.606, *p* = 0.003), while 68% reported that information, services, and programs in the community were inadequate for their children (AOR = 0.211, CI: 0.79–0.562, *p* = 0.002). Over 70% of parents of children with CP identified money as a resource that is usually unavailable or inadequate (AOR = 0.219, CI: 0.080–0.595, *p* = 0.003).

## 4. Discussion

This study is one of the first studies to examine the degree to which children with CP participate in their homes and communities in the Saudi region. The study further looked at factors associated with children with CP’s participation in comparison to typically developing children using a standardized and validated Arabic-language assessment. Our findings show that children with CP have much lower participation levels in activities that are physically demanding than their typically developing peers. Parents of children with CP reported greater difficulties experienced by their children in the community environment related to physically and cognitively demanding activities, as well as in relationships with friends, compared to typically developing children. Lower participation was reported in neighborhood outings, organized physical activities, unsupported classes, religious activities, and gathering with friends in the community. Furthermore, the unavailability and inadequacy of services and supplies supporting children’s participation in the community were perceived by most parents of children with CP as environmental barriers.

Similar findings were found for home participation, where parents of children with CP reported increased difficulties in home tasks related to physical, cognitive, and social activities [23]. Home tasks, such as hobbies, completing chores, and personal care management, have been reported to be lower in children with CP [24].

Our results of lower participation levels in activities that require physical demands in children with CP compared to their typically developing peers are in line with previous studies that reported the same results when using PEM-CY [25]. For example, a significant difference in community participation was found between children with disabilities and typically developing children [25,26]. In addition to participation being significantly lower in children with disabilities, one study noted a lack of environmental support [25,26,27]. The results illustrate some of the barriers that children face in the home and community. Many of these barriers are considered physical barriers, and some are personal barriers according to the ICF model. Our data not only address specific barriers and potential facilitators but also explore parents’ beliefs about their children’s involvement and their desire for a change in their children’s current participation level in both home and community activities. Although the results were very similar to the identified items in both home and community participation, the outcomes showed a desire for change and a low involvement level from the parents’ opinion.

Understanding the environmental barriers and facilitators perceived by parents may provide information on modifiable factors that may promote participation. Few interventions are available to be considered. For instance, the “Pathways and Resources for Engagement and Participation” intervention improves participation in adolescents with physical disabilities by modifying environmental barriers for the individual’s selected activity [26].

Our results reveal that parents require additional support to promote their children’s involvement in the home environment. The rehabilitation plan should be family-centered to illustrate a detailed home program based on available tools to facilitate participation [28,29]. Contacting social services may also be needed to advocate for families and provide them with the equipment necessary to enable the effective participation of children in the home environment [30,31]. In addition, lack of financial support and limited transportation options challenge parents and limit their children’s participation. It has been previously reported that sustainable transportation systems can enhance school attendance and community participation [32]. Parents of children with disabilities require additional resources and financial aid to address their children’s needs. This should be considered when caring for children with CP [33]. In Saudi Arabia, various forms of financial and institutional support are offered for children with disabilities with increased financial support for more severe disabilities with increased resources. Yet, parents of children with disabilities are sometimes unaware of the services available to them or have delayed eligibility due to various reasons (e.g., delays in healthcare provider reports on eligibility). In addition, resources are limited in rural areas compared to cities, which can explain our current results. These factors can contribute to the lack of support and resources that reported by the families participating in our study. Better communication between healthcare providers and families is recommended. Institutional support should also be more accessible. Moreover, to educate parents of children with disabilities about the available resources, recent efforts have been made by special organizations to gather the available services that the children as well as their families can utilize. Publishing these documents would be beneficial to assist parents in accessing all of the needed services. Lastly, communication between governmental and non-governmental agencies should be more organized to meet the goals of the families and their children.

There is a need for a more in-depth recognition of participation and how participation is determined within and between social, family, and environmental contexts and how these elements can work as facilitators or hinderers in both home and community settings [34,35]. Moreover, the lack of a culturally sensitive, child-specific participation assessment contributes to the lack of understanding and research on participation in general and specifically in children with disabilities [36,37,38]. Studies have reported that early learning tasks such as communication, mobility, and interaction in young children with CP improve parents’ perspectives on their children’s participation [9]. Therapists should thus include interventions that target participation when setting up the child’s therapeutic goals [9,39].

The findings of the current study show that 42% of children with CP do not enroll in school. This high percentage is surprising, as public school systems in Saudi Arabia are obligated to provide education to all children and accommodate their needs; therefore, the reasons for the low rate of school attendance should be verified [40]. This finding can be attributed to children being in day care facilities which are not fully inclusive. This finding highlights the need for broader social policies supporting community participation. Our findings are in line with the experiences of some children who face barriers affecting their inclusion in age-appropriate activities such as attending schools in other cities in the Middle East [17,41]. Moreover, school age in Saudi Arabia starts at the age of six years and some of our cohort children were below that age. In addition, most schools’ inclusion criteria are not fully adapted to children with CP GMFCS levels IV and V (22% of our sample size). These can all be contributory factors to our finding of a high percentage of children with CP not being enrolled in schools.

Our findings have the potential to help guide future studies on community and home participation and environmental factors in children with CP, where children experience barriers affecting their participation and inclusion in age-appropriate activities. Studies with larger sample sizes are required to further explore children’s school participation and involvement. Future studies should investigate the relationship between socioeconomic factors and participation in community activities. A larger multi-condition study should be conducted in the future to validate our initial findings. Lastly, for the PEM-CY, it would be recommended to use the original 5 Likert scale system for a more informative understanding of the involvement levels.

Involvement in terms of gathering together with friends in the community was reported to be lower in children with CP than in their typically developing peers, with an extremely high desire among parents to change community activities. This may be a contributing factor to why children with disabilities develop fewer friendships than typically developing children [12]. Moreover, interventions that promote participation are urgently needed and may also ameliorate these obstacles [31].

This study is the first to report on the participation of children with disabilities in the community and at home in Saudi Arabia that used a participation outcome scale measuring both school enrollment and involvement in community activities among school-age children with CP. The strengths of this study include the use of typically developing controls to compare children with CP with typically developing children. In addition, using a validated Arabic version of the PEM-CY, a well-recognized national standardized assessment tool was a strength of this study. Despite the strengths of our study, some limitations exist. The results should be interpreted with caution because of the relatively small sample size. Moreover, the use of only parent-reported instruments potentially limited our results. In fact, we excluded the school domain from the analysis due to the fact that almost half of the children with CP were not enrolled in integrated education programs (daycare) and had limited access to daycare, making it difficult to capture children’s participation levels in schools according to parents’ reports. Additionally, due to the lack of access to contact information of children with CP, randomization was not possible, and the risk of selection bias was therefore present and hard to control.

Current evidence shows that fewer differences in participation between children with motor impairments and typically developing children are observed among young children than among older children [5]. Therefore, it is recommended to start interventions early in preschool, the opportune time to promote participation in children with motor impairment before patterns of reduced participation have been established [42]. With the recent rapid increase in CP registers globally, our study highlights the need for a CP registry to understand the specific characteristics and needs of individuals with CP in Saudi Arabia and identify subgroups of children requiring specific attentions. Establishing such a registry will support the development of appropriate policies and services [43].

## 5. Conclusions

Children with CP participate less in their home and community environments than typically developing children. Limited accessibility to the community further restricts their abilities to participate in education, self-care, and community-based activities. These findings indicate the areas in which further interventions are required to facilitate the community involvement of children with CP. The findings of this study have the potential to guide future research concerning the participation of children with disabilities in school, community, and home activities. Children with CP as well as their caregivers may benefit from ongoing support that promotes these children’s participation.

## Figures and Tables

**Table 1:** Demographic characteristics of the children.

Variable	Children with CP (*n* = 50)	Typically Developing Children (*n* = 50)	*p* Value
**Gender**			0.841
Female	22 (44%)	23 (46%)	
Male	28 (56%)	27 (54%)	
**Age (Mean ± SD)**	10.1 ± 2.426	10.56 ± 2.314	0.3344
**Type of School**			
Public school	16 (32%)	33 (66%)	**<0.001**
Inclusion education	6 (12%)	-	
Private school	7 (14%)	17 (34%)	
Do not attend any school	21 (42%)	-	
**Type of Cerebral Palsy**			-
Spastic hemiplegia	7 (14%)	-	
Spastic diplegia	18 (36%)	-	
Spastic quadriplegia	15 (30%)	-	
Spastic paraplegia	6 (12%)	-	
Ataxia	4 (8%)	-	
**Gross Motor Function Classification System**			
Level I	2 (4%)		
Level II	19 (38%)		
Level III	18 (36%)		
Level IV	8 (16%)		
Level V	3 (6%)		
**Father Education level**			0.448
Elementary	2 (4%)	1 (2%)	
Middle	6 (12%)	4 (8%)	
High school	25 (50%)	22 (44%)	
Higher education	14 (28%)	22 (44%)	
Other *	3 (6%)	1 (2%)	
**Mother Education level**			**0.002**
Elementary	4 (8%)	2 (4%)	
Middle	7 (14%)	18 (36%)	
High school	14 (28%)	22 (44%)	
Higher education	21 (42%)	8 (16%)	
Other	4 (8%)	0	

Data are presented as *n* (%). Note: bold indicates statistical significance at <0.05. * Other: lower than elementary education level.

**Table 2:** Community participation patterns of children with cerebral palsy and typically developing children.

Variable		Children with CP, *n* (%)	Typically Developing Children, *n* (%)	Adjusted Odds Ratio (AOR)	95% CI	Adjusted *p*-Value
Neighborhood outings (e.g., shopping at the store/mall, going to a movie, eating out at a restaurant, visiting the local library/bookstore)	Daily	2 (4)	0	1	-	-
	Weekly	11 (22)	25 (50)	0.105	(0.020–0.539)	**0.007**
	Monthly	24 (48)	22 (44)	0.167	(0.035–0.799)	**0.025**
	Never	13 (26)	3 (6)	Ref	Ref	Ref
Community events (e.g., attending a play, concert, sports game, parade)	Daily	0	1 (2)	1	-	-
	Weekly	2 (4)	2 (4)	2.873	(0.283–29.135)	0.372
	Monthly	5 (10)	16 (32)	0.300	(0.079–1.137)	0.077
	Never	43 (86)	31 (62)	Ref	Ref	Ref
Organized physical activities (e.g., sports teams or classes such as baseball, hockey, martial arts, dance, horseback riding, swimming, gymnastics)	Daily	1 (2)	12 (24)	0.024	(0.005–0.671)	**0.002**
	Weekly	8 (16)	15 (30)	0.184	(0.054–0.628)	**0.007**
	Monthly	1 (2)	6 (12)	0.059	(0.002–256)	**0.022**
	Never	40 (80)	17 (34)	Ref	Ref	Ref
Unorganized physical activities (e.g., walking in nature, riding a bike, skiing, skateboarding, playing hide-and-seek or running, playing basketball)	Daily	4 (8)	4 (8)	0.876	(0.141–5.436)	0.212
	Weekly	8 (16)	15 (30)	0.252	(0.069–0.924)	**0.038**
	Monthly	8 (16)	13 (26)	0.460	(0.136–1.559)	0.887
	Never	30 (60)	18 (36)	Ref	Ref	Ref
Unsupported classes by the school (e.g., music, art, computer languages)	Daily	5 (10)	12 (24)	0.251	(0.059–1.059)	0.060
	Weekly	6 (12)	17 (34)	0.132	(0.036–0.480)	**0.002**
	Monthly	4 (8)	6 (12)	0.180	(0.032–0.994)	**0.049**
	Never	35 (70)	15 (30)	Ref	Ref	Ref
Religious or spiritual gatherings and activities (e.g., attending places of worship, religion classes, groups)	Daily	7 (14)	23 (46)	0.149	(0.048–0.463)	**0.001**
	Weekly	3 (6)	8 (16)	0.197	(0.038–1.026)	0.054
	Monthly	2 (4)	2 (4)	0.235	(0.012–4.436)	0.334
	Never	38 (76)	17 (34)	Ref	Ref	Ref
Getting together with friends in the community (e.g., hanging out, informal gatherings outside of home or school, BBQ, going out on a date)	Daily	4 (8)	9 (18)	0.047	(0.004–0.619)	**0.020**
	Weekly	22 (44)	29 (58)	0.071	(0.007–0.688)	**0.022**
	Monthly	11 (22)	11 (22)	0.061	(0.005–0.699)	**0.025**
	Never	13 (26)	1 (2)	Ref	Ref	Ref
Overnight visits or trips (e.g., sleepovers, camp, vacations)	Daily	0	5 (10)	1	-	-
	Weekly	4 (8)	2 (4)	2.407	(0.310–18.659)	0.401
	Monthly	8 (16)	11 (22)	0.781	(0.250–2.434)	0.669
	Never	38 (76)	32 (64)	Ref	Ref	Ref

Note: bold indicates statistical significance at <0.05.

**Table 3:** Home participation patterns of children with cerebral palsy and typically developing children.

Variable		Children with CP, *n* (%)	Typically Developing Children, *n* (%)	Adjusted Odds Ratio (AOR)	95% CI	Adjusted *p*-Value
Computer and video games	Daily	37 (74)	39 (78)	Ref	Ref	Ref
	Weekly	3 (6)	10 (20)	0.177	(0.026–1.193)	0.075
	Monthly	9 (18)	1 (2)	14.371	(1.468–140.730)	**0.022**
	Never	1 (2)	0	1	-	-
Arts, crafts, music, and hobbies (e.g., participating in arts and crafts, listening to music, playing an instrument, collecting, reading for leisure, cooking for fun)	Daily	6 (12)	21 (42)	Ref	Ref	Ref
	Weekly	18 (36)	21 (42)	2.536	(0.686–9.375)	0.163
	Monthly	19 (38)	3 (6)	16.420	(3.156–85.443)	**0.001**
	Never	7 (14)	5 (10)	2.827	(0.515–15.501)	0.231
Getting together with other people (e.g., interacting with peers, family, other houseguests) OR Socializing using technology (e.g., telephone, computer)	Daily	24 (48)	34 (68)	Ref	Ref	Ref
	Weekly	8 (16)	10 (20)	1.392	(0.407–4.764)	0.286
	Monthly	14 (28)	4 (8)	4.598	(1.138–18.575)	**0.032**
	Never	4 (8)	2 (4)	4.353	(0.606–31.242)	0.144
Household chores (e.g., unloading/loading the dishwasher, cleaning room or other areas of the house, cooking, taking out the garbage, setting the table, caring for the household pet)	Daily	2 (4)	15 (30)	Ref	Ref	Ref
	Weekly	7 (14)	27 (54)	2.344	(0.313–17.551)	0.407
	Monthly	39 (78)	0	1	-	-
	Never	2 (4)	8 (16)	3.208	(0.252–40.837)	0.369
Personal care management (e.g., getting dressed, choosing clothing, brushing hair or teeth, applying makeup)	Daily	21 (42)	45 (90)	Ref	Ref	Ref
	Weekly	7 (14)	5 (10)	2.413	(0.585–9.961)	0.223
	Monthly	21 (42)	0	1	-	-
	Never	1 (2)	0	1	-	-
Homework (e.g., daily reading, homework assignments, school projects)	Daily	45 (90)	45 (90)	Ref	Ref	Ref
	Weekly	5 (10)	5 (10)	1.004	(0.213–4.745)	0.996
	Monthly	0	0	-	-	-
	Never	0	0	-	-	-

Note: bold indicates statistical significance at <0.05.

**Table 4:** Perceived supportiveness of the home environment with the study subjects.

Variable		Children with CP, *n* (%)	Typically Developing Children, *n* (%)	Adjusted Odds Ratio (AOR)	95% CI	Adjusted *p*-Value
The physical layout or amount of space and furniture in your home	Usually helps	11 (22)	26 (52)	0.166	(0.052–0.527)	**0.002**
	Usually makes hard	12 (24)	11 (22)	0.378	(0.107–1.337)	0.131
	Not an issue	27 (54)	13 (26)	Ref	Ref	Ref
The sensory qualities of the home environment (e g. amount and/or type of sound, light, temperature, textures of objects)	Usually helps	25 (50)	33 (66)	0.480	(0.156–2.301)	0.199
	Usually makes hard	9 (18)	7 (14)	0.513	(0.115–2.301)	0.384
	Not an issue	16 (32)	10 (20)	Ref	Ref	Ref
The physical demands of typical activities in the home (e.g., strength, endurance, coordination)	Usually helps	23 (46)	37 (74)	0.407	(0.113–1.466)	0.169
	Usually makes hard	17 (34)	7 (14)	1.694	(0.361–7.958)	0.504
	Not an issue	10 (20)	6 (12)	Ref	Ref	Ref
The cognitive demands of typical activities in the home (e.g., concentration, attention, problem solving)	Usually helps	22 (44)	40 (80)	0.116	(0.020–0.685)	**0.017**
	Usually makes hard	17 (34)	8 (16)	0.398	(0.060–2.693)	0.345
	Not an issue	11 (22)	2 (4)	Ref	Ref	Ref
The social demands of typical activities in the home (e.g., communication, interacting with others)	Usually helps	34 (68)	47 (94)	0.075	(0.007–0.771)	**0.029**
	Usually makes hard	6 (12)	2 (4)	0.175	(0.009–3.235)	0.242
	Not an issue	10 (20)	1 (2)	Ref	Ref	Ref

Note: bold indicates statistical significance at <0.05.

**Table 5:** Perceived supportiveness of the community environment.

Variable		Children with CP, *n* (%)	Typically Developing Children, *n* (%)	Adjusted Odds Ratio (AOR)	95% CI	Adjusted *p*-Value
The physical layout or amount of space outside and inside buildings (e.g., distances to stores, presence of sidewalks, availability of ramps or elevators)	Usually helps	23 (46)	24 (48)	1.075	-	0.905
	Usually makes it hard	16 (32)	15 (30)	1.124	(0.318–3.979)	0.856
	Not an issue	11 (22)	11 (22)	Ref	Ref	Ref
The sensory qualities of community settings (e.g., noise, crowds, lighting)	Usually helps	10 (20)	10 (20)	0.982	(0.248–3.892)	0.979
	Usually makes it hard	26 (52)	29 (58)	0.695	(0.236–2.048)	0.509
	Not an issue	14 (28)	11 (22)	Ref	Ref	Ref
The physical demands of typical activities (e.g., strength, endurance, coordination)	Usually helps	19 (38)	34 (68)	0.907	(0.245–3.357)	0.884
	Usually makes it hard	22 (44)	7 (14)	3.593	(0.787–16.409)	0.099
	Not an issue	9 (18)	9 (18)	Ref	Ref	Ref
The cognitive demands of typical activities (e.g., focus, attention, problem solving)	Usually helps	25 (50)	40 (80)	0.834	(0.153–4.537)	0.834
	Usually makes it hard	20 (40)	6 (12)	2.618	(0.399–17.141)	0.315
	Not an issue	5 (10)	4 (8)	Ref	Ref	Ref
The social demands of typical activities (e.g., communication, interaction with others)	Usually helps	31 (62)	41 (82)	1.451	(0.273–7.707)	0.662
	Usually makes it hard	14 (28)	5 (10)	2.867	(0.420–19.589)	0.282
	Not an issue	5 (10)	4 (8)	Ref	Ref	Ref
Child’s relationship with their friends	Usually helps	32 (64)	46 (92)	0.230	(0.040–1.338)	0.102
	Usually makes it hard	7 (14)	2 (4)	0.577	(0.053–6.279)	0.652
	Not an issue	11 (22)	2 (4)	Ref	Ref	Ref
The attitudes and actions of community members toward your child (e.g., shop owner, supervisor, trainers, other families)	Usually helps	25 (50)	35 (70)	0.741	(0.239–2.303)	0.605
	Usually makes it hard	12 (24)	6 (12)	1.835	(0.401- 8.388)	0.434
	Not an issue	13 (26)	9 (18)	Ref	Ref	Ref
External weather conditions (e.g., climate, temperature)	Usually helps	18 (36)	21 (42)	0.480	(0.154–1.497)	0.206
	Usually makes it hard	14 (28)	17 (34)	0.395	(0.123–1.266)	0.118
	Not an issue	18 (36)	12 (24)	Ref	Ref	Ref
Safety in society (e.g., traffic, crime, violence)	Usually helps	18 (36)	17 (34)	0.834	(0.482–1.445)	0.517
				0.678	(0.197–2.338)	0.539
	Usually makes it hard	14 (28)	25 (50)	0.261	(0.077–0.893)	**0.032**
	Not an issue	18 (36)	8 (16)	Ref	Ref	Ref

Note: bold indicates statistical significance at <0.05.

## Data Availability

The data that support the findings of this study are available on reasonable request from the corresponding author, but restrictions apply due to privacy and ethical restrictions.

## Supplementary Materials

The following supporting information can be downloaded at: https://www.mdpi.com/article/10.3390/pediatric17010017/s1, Table S1: Community participation involvement of children with cerebral palsy and typically develop children; Table S2: Community participation desire to change of children with cerebral palsy typically develop children; Table S3: Home participation involvement of children with cerebral palsy and typically develop children; Table S4: Home participation desire to change of children with cerebral palsy and typically develop children; Table S5: Relationship between Perceived availability and adequacy of resources with the study subjects; Table S6: Relationship between perceived availability and adequacy of resources.
