# Supplementary material for: Measuring Community and Home Participation and Environmental Factors in Children with Cerebral Palsy
Source: Pediatr Rep. 2025 Feb 7;17(1):17. doi: 10.3390/pediatric17010017 (PMC11858706; doi:10.3390/pediatric17010017)
Supplement: Supplementary file 1 [file pediatrrep-17-00017-s001.zip › pediatrrep-3418336-supplementary.pdf]

Supplementary

S1: Community participation involvement of children with cerebral palsy and typically develop children

**Community participation items**		**Involvement**				
		**Children with CP N(%)**	**Typically develop children N(%)**	**Adjusted Odds Ratio (AOR)**	**95% CI**	**Adjusted P-value**
Neighborhood outings (e.g. shopping at the store/mall, going to a movie, eating out at a restaurant, visiting the local library/ bookstore)	High	12(24)<br>13 (26)	42(84)<br>25 (50)	0.497	(0.191 - 1.296)	0.153
	Low	15(30)<br>37 (74)	5(10)<br>25 (50)	Ref	Ref	Ref
Community events (e.g. attending a play, concert, sports game, parade)	High	2(4)<br>2 (4)	7(14)<br>3 (6)	2.413	(0.304 - 19.137)	0.404
	Low	5(10)<br>48 (96)	12(24)<br>47 (94)	Ref	Ref	Ref
Organized physical activities (e.g. sports teams or classes such as baseball, hockey, martial arts, dance, horseback riding, swimming gymnastics)	High	3(6)<br>9 (18)	22(44)<br>28 (56)	0.145	(0.049 – 0.431)	**0.001**
	Low	7(14)<br>41 (82)	10(20)<br>22 (44)	Ref	Ref	Ref
Unorganized physical activities (e.g. walking in nature, riding a bike, skiing, skateboarding, playing hide-and-seek or running, running playing like basketball)	High	6(12)<br>12 (24)	15(30)<br>19 (38)	0.473	(0.167 – 1.344)	0.160
	Low	14(28)<br>38 (76)	17(34)<br>31 (62)	Ref	Ref	Ref
Unsupported Classes by the school (e.g. music, art, computer languages)	High	5(10)<br>11 (22)	22(44)<br>29 (58)	0.238	(0.088 - 0.649)	**0.005**
	Low	10(20)<br>39 (78)	13(26)<br>21 (42)	Ref	Ref	Ref
Religious or spiritual gatherings and activities(e.g., attending places of worship, religion classes, groups)	High	3(6)<br>10 (20)	21(42)<br>32 (64)	0.171	(0.061 – 0.481)	**0.001**
	Low	9(18|)<br>40 (80)	12(24)<br>18 (36)	Ref	Ref	Ref

Getting together with friends in the community(e.g., hanging out, informal gatherings outside of home or school, BBQ, going out on a date)	High	24(48)<br>26 (52)	44(88)<br>38 (76)	0.473	(0.175 – 1.280)	0.140
	Low	13(26)<br>24 (48)	5(10)<br>12 (24)	Ref	Ref	Ref
Overnight visits or trips(e.g., sleepovers camp, vacations	High	4(8)<br>4 (8)	8(16)<br>7 (14)	0.557	(0.126 – 2.456)	0.440
	Low	8(16)<br>46 (92)	10(20)<br>43 (86)	Ref	Ref	Ref

Note: bold indicates statistically significant at <0.05.

S2: Community participation desire to change of children with cerebral palsy typically develop children

**Community participation items**		**Desires to change**		**Adjusted Odds Ratio (AOR)**	**95% CI**	**Adjusted P-value**
		Children with CP N(%)	Typically develop children N(%)			
Neighborhood outings<br>(e.g. shopping at the store/mall, going to a movie, eating out at a restaurant, visiting the local library/ bookstore)	Yes	24(48)	12(24)	2.188	(0.823 - 5.815)	0.116
	No	26(52)	38(76)	Ref	Ref	Ref
Community events<br>(e.g. attending a play, concert, sports game, parade)	Yes	24(48)	17(34)	1.783	(0.695 - 4.574)	0.229
	No	26(52)	33(66)	Ref	Ref	Ref

Organized physical activities (e.g. sports teams or classes such as baseball, hockey, martial arts, dance, horseback riding, swimming gymnastics)	Yes	31(62)	19(38)	2.210	(0.877 – 5.570)	
	No	19(38)	31(62)	Ref	Ref	Ref
Unorganized physical activities (e.g. walking in nature, riding a bike, skiing, skateboarding, playing hide-and-seek or running, running playing like basketball)	Yes	31(62)	21(42)	1.960	(0.767 – 5.008)	0.160
	No	19(38)	29(58)	Ref	Ref	Ref
Unsupported Classes by the school (e.g. music, art, computer languages)	Yes	30(60)	23(46)	1.353	(0.537 – 3.407)	0.637
	No	20(40)	27(54)	Ref	Ref	Ref
Religious or spiritual gatherings and activities (e.g., attending places of worship, religion classes, groups)	Yes	31(62)	18(36)	3.227	(1.218 – 8.550)	**0.018**
	No	19(38)	32(64)	Ref	Ref	Ref
Getting together with friends in the community(e.g., hanging out, informal gatherings outside of home or school, BBQ, going out on a date)	Yes	27(54)	13(26)	3.063	(1.159 – 8.100)	**0.024**
	No	23(46)	37(74)	Ref	Ref	Ref
Overnight visits or trips (e.g., sleepovers camp, vacations	Yes	14(28)	8(16)	1.770	(0.577 – 5.427)	0.318
	No	36(72)	42(84)	Ref	Ref	Ref

Note: bold indicates statistically significant at <0.05.

S3: Home participation involvement of children with cerebral palsy and typically develop children

**Home participation items**		**Involvement**				
		Children with CP N(%)	Typically develop children N(%)	Adjusted Odds Ratio (AOR)	95% CI	Adjusted P-value
Computer and video games	High	34(68)<br>40 (80)	39(78)<br>49 (98)	0.052	(0.006 – 0.491)	**0.010**
	Low	15(30)<br>10 (20)	11(22)<br>1 (2)	Ref	Ref	Ref
Arts, crafts, music, and hobbles<br>(e.g, doing arts and crafts, listening to music, playing an instrument, collecting. reading for leisure, cooking for fun)	High	15(30)<br>24 (48)	30(60)<br>42 (84)	0.230	(0.081 – 0.653)	**0.006**
	Low	28(56)<br>26 (52)	15(30)<br>8 (16)	Ref	Ref	Ref
Getting together with other people<br>(e.g., interacting with peers, family, other houseguests)<br>OR<br>Socializing using technology<br>(e.g., telephone, computer)	High	26(52)<br>32 (64)	31(62)<br>44 (88)	0.243	(0.075 – 0.789)	**0.019**
	Low	20(40)<br>18 (36)	17(34)<br>6 (12)	Ref	Ref	Ref
Household chores<br>(e.g., unloading/loading the dishwasher, cleaning room or other areas of the house. cooking, taking out the garbage, setting the table, caring for household pet)	High	6(12)<br>9 (18)	18(36)<br>42 (84)	0.032	(0.009 – 0.118)	**0.000**
	Low	42(84)<br>41 (82)	32(64)<br>8 (16)	Ref	Ref	Ref
Personal care management<br>(e.g- getting dressed, choosing clothing. brushing hair or teeth, applying makeup)	High	20(40)<br>28 (56)	36(72)<br>50 (100)	1	-	-
	Low	29(58)<br>22 (44)	14(28)<br>0	Ref	Ref	Ref
Homework<br>(e.g., daily reading, homework assignments, school projects)	High	23(46)<br>50 (100)	23(46)<br>50 (100)	1	-	-
	Low	27(54)	27(54)	Ref	Ref	Ref

Note: bold indicates statistically significant at <0.05.

S4: Home participation desire to change of children with cerebral palsy and typically develop children

Home participation items		Desires to change				
		**Children with CP N(%)**	**Typically develop children N(%)**	**Adjusted Odds Ratio (AOR)**	**95% CI**	**Adjusted P-value**
Computer and video games	Yes	35(70)	40(80)	0.553	(0.191 – 1.600)	0.274
	No	15(30)	10(20)	Ref	Ref	Ref
Arts, crafts, music, and hobbles (e.g, doing arts and crafts, listening to music, playing an instrument, collecting. reading for leisure, cooking for fun)	Yes	34(68)	17(34)	3.951	(1.538 – 10.146)	**0.004**
	No	16(32)	33(66)	Ref	Ref	Ref
Getting together with other people (e.g., interacting with peers, family, other houseguests) <u>OR</u> Socializing using technology (e.g., telephone, computer)	Yes	28(56)	25(50)	1.322	(0.529 – 3.299)	0.550
	No	22(44)	25(50)	Ref	Ref	Ref
Household chores (e.g., unloading/loading the dishwasher, cleaning room or other areas of the house. cooking, taking out the garbage, setting the table, caring for household pet)	Yes	33(66)	26(52)	1.476	(0.597 - 3.654)	0.399
	No	17(34)	24(48)	Ref	Ref	Ref
Personal care management (e.g- getting dressed, choosing clothing. brushing hair or teeth, applying makeup)	Yes	32(64)	13(26)	4.544	(1.646 - 12.539)	**0.003**
	No	18(36)	37(74)	Ref	Ref	Ref
Homework	Yes	24(48)	0(0)	1	-	-

(e.g., daily reading, homework assignments, school projects)	No	26(52)	50(100)	Ref	Ref	Ref

Note: bold indicates statistically significant at <0.05.

S5: Relationship between Perceived availability and adequacy of resources with the study subjects

Particulars	Frequency (Percentage)				Adjusted Odds Ratio (AOR)	95% CI	Adjusted P-value
	Yes		No				
	Children with CP N(%)	Typically develop children N(%)	Children with CP N(%)	Typically develop children N(%)			
Supplies in the home (e.g., sports equipment, crafts supplies, reading materials, assistive devices or technology, picture or word schedules)	24(48)	49(98)	26(52)	1(2)	0.018	(0.002 – 0.170)	**<0.001**
Do you (and your family) have enough time to support your child's participation at home?	36(72)	44(88)	14(28)	6(12)	0.332	(0.099 – 1.112)	0.075
Do you (and your family) have enough money to support your child's participation at home?	20(40)	45(90)	30(60)	5(10)	0.067	(0.019 – 0.230)	**<0.001**

Note: bold indicates statistically significant at <0.05.

S6: Relationship between perceived availability and adequacy of resources

Particulars	Frequency (Percentage)				Adjusted Odds Ratio (AOR)	95% CI	Adjusted P-value
	Yes		No				
	Children with CP N(%)	Typically develop children N(%)	Children with CP N(%)	Typically develop children N(%)			
Personal transportation to attend social activities(e.g. family car, bicycle)	22(44)	40(80)	28(56)	10(20)	0.220	(0.796 - 0.606)	**0.003**
Programs and services (e.g. comprehensive sports programs, personal assistants)	14(28)	33(66)	36(68)	17(34)	0.211	(0.079 – 0.562)	**0.002**
Financial ability to support the child's participation in society	21(42)	39(78)	29(58)	11(22)	0.219	(0.080 – 0.595)	**0.003**

Note: bold indicates statistically significant at <0.05.
